# QT Interval Monitoring with Handheld Heart Rhythm ECG Device in COVID-19 Patients

**DOI:** 10.5334/gh.916

**Published:** 2021-06-08

**Authors:** Carlos Minguito-Carazo, Julio Echarte-Morales, Tomás Benito-González, Samuel del Castillo-García, Miguel Rodríguez-Santamarta, Enrique Sánchez-Muñoz, Clea González Maniega, Rubén García-Bergel, Paula Menéndez-Suárez, Silvia Prieto-González, Carmen Palacios-Echavarren, Javier Borrego-Rodríguez, Guisela Flores-Vergara, Ignacio Iglesias-Garriz, Felipe Fernández-Vázquez

**Affiliations:** 1 Department of Cardiology, University Hospital of León, León, ES

**Keywords:** COVID-19, QTc, ventricular arrhythmia, KardiaMobile-6L, handheld ECG

## Abstract

**Background:**

QTc prolongation is an adverse effect of COVID-19 therapies. The use of a handheld device in this scenario has not been addressed.

**Objectives:**

To evaluate the feasibility of QTc monitoring with a smart device in COVID-19 patients receiving QTc-interfering therapies.

**Methods:**

Prospective study of consecutive COVID-19 patients treated with hydroxychloroquine ± azithromycin ± lopinavir-ritonavir. ECG monitoring was performed with 12-lead ECG or with KardiaMobile-6L. Both registries were also sequentially obtained in a cohort of healthy patients. We evaluated differences in QTc in COVID-19 patients between three different monitoring strategies: 12-lead ECG at baseline and follow-up (A), 12-lead ECG at baseline and follow-up with the smart device (B), and fully monitored with handheld 6-lead ECG (group C). Time needed to obtain an ECG registry was also documented.

**Results:**

One hundred and eighty-two COVID-19 patients were included (A: 119(65.4%); B: 50(27.5%); C: 13(7.1%). QTc peak during hospitalization did significantly increase in all groups. No differences were observed between the three monitoring strategies in QTc prolongation (p = 0.864). In the control group, all but one ECG registry with the smart device allowed QTc measurement and mean QTc did not differ between both techniques (p = 0.612), displaying a moderate reliability (ICC 0.56 [0.19–0.76]). Time of ECG registry was significantly longer for the 12-lead ECG than for handheld device in both cohorts (p < 0.001).

**Conclusion:**

QTc monitoring with KardiaMobile-6L in COVID-19 patients was feasible. Time of ECG registration was significantly lower with the smart device, which may offer an important advantage for prevention of virus dissemination among healthcare providers.

## 1. Introduction

To date, several therapies have been empirically used to treat Coronavirus disease 2019 (COVID-19). Most of these therapies are associated to a prolongation of corrected QT interval (QTc) as a potential side effect and, consequently, to an increased risk of ventricular arrythmias (VA) and sudden cardiac death (SCD) [1]. Therefore, QTc monitoring should be considered while these treatments are being administered.

Conventional 12-lead electrocardiogram (ECG) using limb leads is commonly used to measure QTc [2]. However, in the scenario of COVID-19, the performance of a 12-lead ECG entails close contact with the patient and a major risk of exposure to the virus for healthcare workers. In order to prevent virus dissemination, the use of smart mobile handheld ECG devices for QTc monitoring has emerged as an appealing alternative. With this purpose, AliveCor (AliveCor, San Francisco, CA, USA) has recently received clearance from the Food and Drug Administration (FDA) for the use of KardiaMobile-6L device to monitor the ECG in COVID-19 patients treated with drugs interfering with QTc. However, no data is currently available assessing QTc monitoring with this device in this setting. The aim of this study was to evaluate the feasibility of QTc monitoring with a heart rhythm smart device in COVID-19 patients receiving QTc interfering therapies during hospitalization.

## 2. Methods

### 2.1 Study population

**Figure 1:**
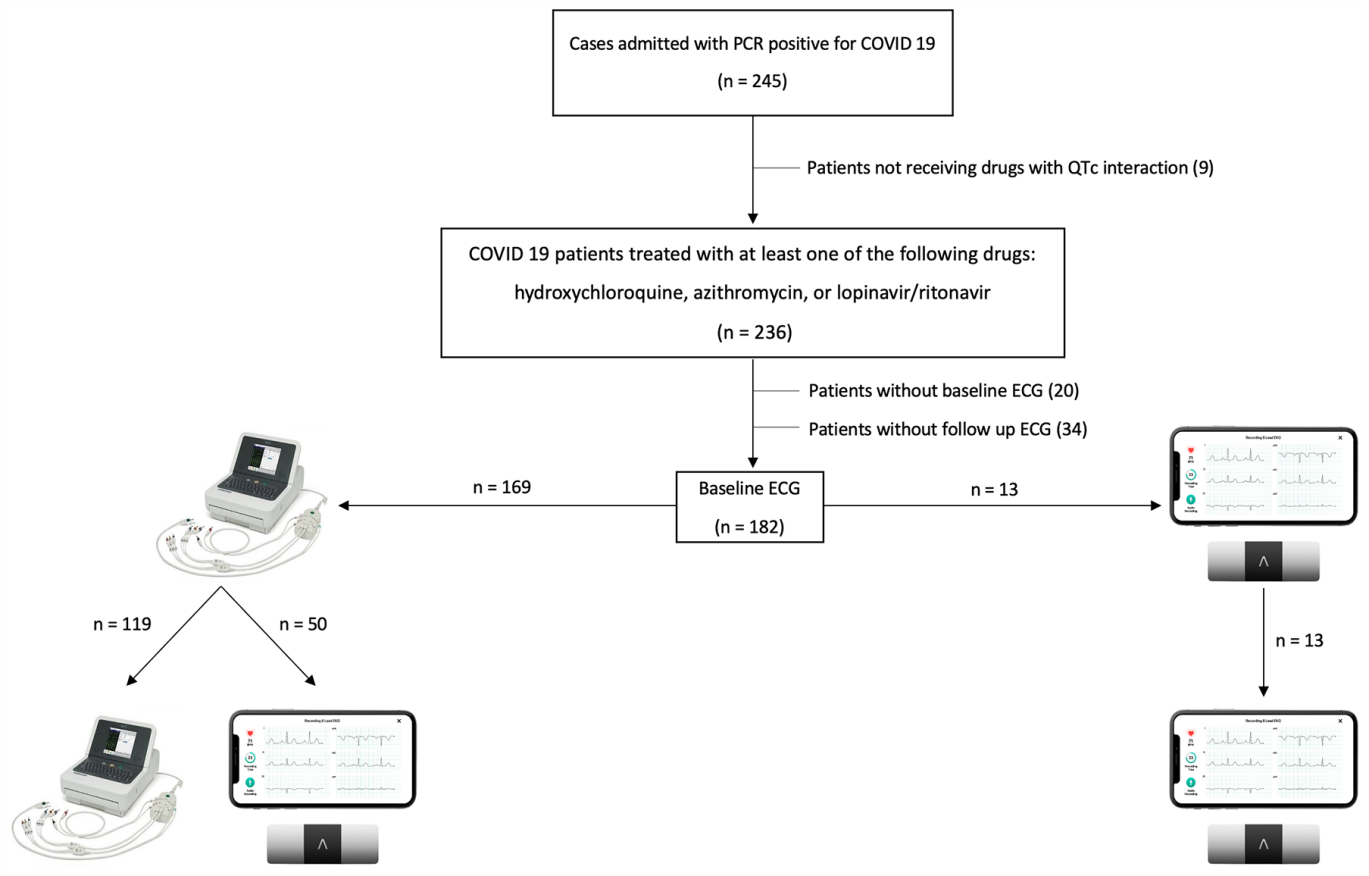
Study flow chart.

A prospective, observational, single-center study of consecutive patients admitted to our institution for confirmed COVID-19 pneumonia since 31 March 2020 to 5 May 2020 was conducted. We included patients with a positive COVID-19 nasopharyngeal polymerase chain reaction (PCR) receiving at least one dose of one of the following QTc-prolonging drugs: azithromycin (AZ), hydroxychloroquine and/or lopinavir-ritonavir. Patients in which a baseline ECG and at least one follow-up ECG during active treatment were not carried out were excluded from the analysis (Figure 1).

In order to safely confirm feasibility of QTc measurement with KardiaMobile-6L compared to conventional 12-lead ECG, both registries were sequentially obtained in a consecutive cohort of healthy patients with negative COVID-19 PCR as a control group.

### 2.2 Study procedures

#### 2.2.1. Medical Treatment

The choice of treatment in each patient was based on physician’s criteria and local guidelines. Changes in medication, including those related to iatrogenic QTc prolongation, were individualized.

#### 2.2.2. Electrocardiographic Monitoring

As proposed by Giudicessi, et al., an ECG was performed in all patients receiving at least one QTc-prolonging medication before any drug was started and repeated at 48 hours and 96 hours, and every two to three days while continuing any of these therapies [3]. ECG recordings during admission were carried out using 12-lead conventional ECG or KardiaMobile-6L device depending on its availability (the smart device was progressively implemented in daily practice in most hospitalization wards) and the clinical condition of the patient (since 6-lead ECG with the handheld device could only be obtained in relatively stable conscious subjects). The decision to use one or other ECG monitoring device was left to physician’s discretion. Patients were allocated in three groups for the analysis depending on the device used to measure the QTc at baseline and during follow-up within the admission (Figure 1): A) conventional 12-lead ECG before and after COVID-19 therapies were started; B) conventional 12-lead ECG at admission, followed up with handheld device; and C) fully monitored with heart rhythm smart device.

**Figure 2:**
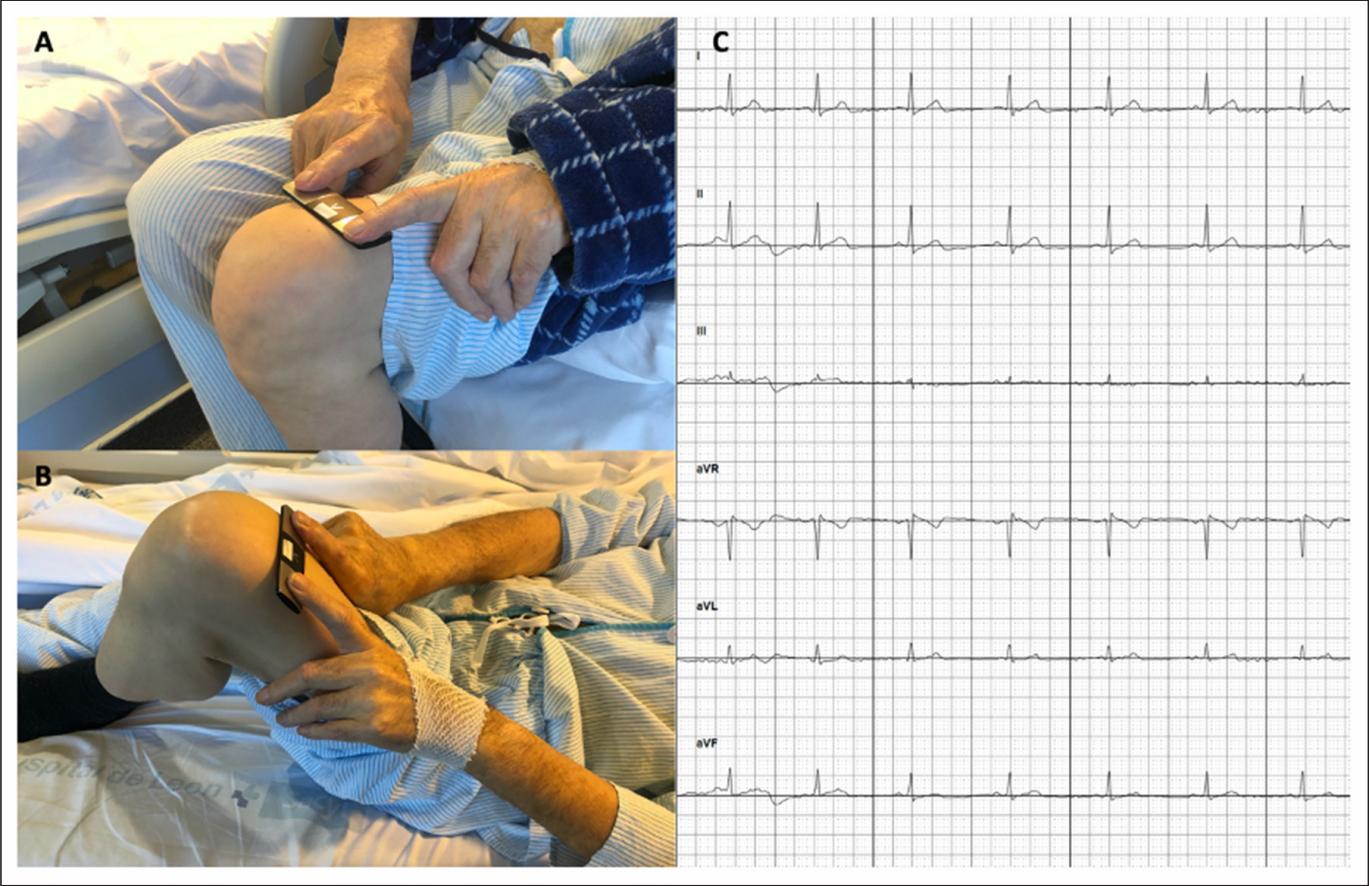
ECG register with KardiaMobile-6L.

Conventional 12-lead ECG was obtained as in common clinical practice, although the equipment needed to be fully cleaned and disinfected before leaving the room afterwards. To register a 6-lead ECG with the handheld device, the patient was told to be sit and place the device on the bare skin of his left leg (at the knee or the ankle) holding his thumbs on the top of two electrodes for 30 seconds (Figure 2A). If the patient could not sit, the ECG was recorded while lying down, flexing the left knee closer to the body (Figure 2B). Alternatively, 1-lead ECG (lead I) could be obtained only with two fingers in contact with the electrodes. The ECG registry was wirelessly transmitted to a tablet outside the patient’s room and digitally uploaded by a dedicated app to a secure server. Once the device was used, the electrodes were cleaned spraying with an alcohol-based sanitizer and wiping with a soft cloth before being used in another patient. Since ECG recording with the handheld device requires the active collaboration of the patient, standard 12-lead ECG was carried out among those in worse clinical status. Similarly, if an accurate registry could not be obtained for whatever reason, a conventional 12-lead ECG was performed.

In order to assess healthcare workers exposure, the duration of the ECG recording was documented for each ECG registry (when possible) and compared between both techniques. Procedural time accounted since the healthcare worker entered into the patient’s room to the disinfection of the device (either conventional 12-lead ECG or KardiaMobile-6L) once the register was performed. Similarly, ECG recording time was addressed in the healthy control cohort (since the patient lied down in bed until postprocedural disinfection measurements were completed).

#### 2.2.3 QTC Measurement

ECGs records were reviewed and interpreted by at least one of two cardiologists (C.M.C. and J.E.M). QTc interval was calculated using Bazett’s formula in leads II or V5 in the 12-lead ECG and in lead II when using the handheld 6-lead ECG [4]. If these leads did not provide an accurate end of the T wave, I and aVL were preferably used as an alternative, although QTc measurement in any other lead was permitted. For patients with a wide QRS from either ventricular pacing or left/right bundle branch block the excess correction method was used [5]. In patients with atrial fibrillation (AF), QTc was estimated as the mean of three to five beats. To ensure the integrity of the data, none of the ECG interpreters knew the therapy administered to the patient.

The longest QTc measured after COVID-19 drugs were started was considered as peak QTc during admission and compared with the QTc at baseline to evaluate QTc prolongation. Patients with a peak QTc ≥ 500 ms and those with an increase in the QTc ≥ 60 ms from baseline values were considered at high risk for VA [6].

With the aim to evaluate the feasibility of QTc measurement with 6-lead handheld device compared to conventional 12-lead ECG, QTc was also blindly addressed in the healthy control cohort.

### 2.3 Study endpoints

The main objective of this study was to evaluate differences in QTc monitoring in COVID-19 patients receiving QTc-prolonging drugs between conventional 12-lead ECG and 6-lead heart rhythm handheld device. Time needed to obtain an ECG registry with both devices was set up as a secondary endpoint.

### 2.4 Data collection

All data were collected using standardized report forms including demographic features, medical history, baseline clinical characteristics, biochemical and ECG findings, medical treatment during admission and in-hospital clinical outcomes. CURB65 (Confusion, BUN [Blood Urea Nitrogen] > 19 mg/dl, Respiratory rate < 30, Systolic blood pressure < 90 mmHg or diastolic blood pressure < 60 mmHg, Age > 65 years) and Tisdale scores were estimated at admission. The study was approved by the local Ethics Committee (reference 2059) and adhered to the principles outlined in the Declaration of Helsinki. Individual informed consent was obtained from participants.

### 2.5 Statistical analysis

Continuous variables were summarized as mean ± standard deviation (SD) or as medians and interquartile range (IQR) and were compared between groups using ANOVA or Kruskall Wallis tests depending on normality. Differences in the time of ECG recording between devices and changes in QTc from baseline to peak were compared using paired or unpaired Student T tests or the non-parametric Wilcoxon rank sum tests if the normal distribution the variables could not be demonstrated. Categorical variables were described as percentages and compared using Chi-square or Fisher exact tests according to expected frequency over or below five, respectively. Derangement from the normal distribution was assessed with the Shapiro-Wilk test. A two-sided p-value < 0.05 was regarded as statistically significant. Statistical analyses were performed using STATA software version 15.1.

## 3. Results

### 3.1 Patient population

During the entire recruiting period 245 patients with positive COVID-19 PCR were identified (Figure 1). Finally, 182 (66.5 ± 14.6 years old, male 58.2%) COVID-19 patients were included for the analysis: 119 (65.4%) had a conventional 12-lead ECG before and after COVID-19 therapies were started (group A); 50 (27.5%) had a 12-lead ECG at admission and were followed up with a handheld device (group B); and 13 (7.1%) were fully monitored with heart rhythm smart device (group C).

Baseline characteristics of the three groups are displayed in Table 1. Patients that were fully monitored with conventional 12-lead ECG had a higher proportion of patients with a CURB65 score ≥ 2 at admission than those that were followed up with the handheld device (p = 0.001) (p = 0.033). There were 73 patients with a CURB65 score ≥ 2. From them, 76.7% were monitored only with a 12-lead ECG (group B), 19.2% with a basal 12-lead ECG and then with a handheld device and 4.1% were fully monitored with the handheld device (group C). No other significant differences in baseline features were found between groups. Most patients were on two or more QTc interfering drugs, more frequently in the group monitored with KardiaMobile-6L (p = 0.049), albeit no significant differences in the Tisdale score were observed between groups (p = 0.819).

**Table 1:** Baseline characteristics of the patients.

Variable	Total (n = 168)	12-lead ECG (basal and follow-up) (n = 119)	12-lead ECG (basal) and Handheld device (follow-up) (n = 50)	Handheld device (basal and follow-up) (n = 13)	p-value

Age (years), mean ± SD	66.5 ± 14.6	67.4 ± 14.7	64.5 ± 14.1	66.1 ± 16.0	0.342
Male, n (%)	106 (58.2)	75 (63.0)	22 (44.0)	9 (69.2)	0.051
Hypertension, n (%)	86 (47.3)	59 (49.6)	23 (46.0)	4 (30.8)	0.426
Diabetes, n (%)	38 (20.9)	22 (18.5)	12 (24.0)	4 (30.8)	0.416
Ischemic heart disease, n (%)	13 (7.1)	10 (8.4)	3 (6.0)	0 (0)	0.717
Chronic kidney disease, n (%)	36 (19.8)	23 (19.3)	11 (22.0)	2 (15.4)	0.869
Length of stay (days), mean ± SD	10.2 ± 5.3	10.1 ± 5.5	10.7 ± 5.2	9 ± 4.5	0.314
Serum creatinine (mg/dl), mean ± SD	1.26 ± 1.3	1.31 ± 1.4	1.02 ± 0.4	1.70 ± 2.4	0.284
Loop diuretic in-hospital, n (%)	31 (17.1)	22 (18.5)	8 (16.3)	1 (7.7)	0.730
CURB 65 at admission ≥ 2 (%)	73 (40.6)	56 (47.5)	14 (28.0)	3 (25.0)	0.033
Tisdale score at admission, mean ± SD	8.1 ± 2.4	8.2 ± 2.4	7.9 ± 2.6	8.2 ± 2.4	0.819
≥2 QTc prolonging drug, n (%)	175 (96.2)	117 (98.3)	45 (90.0)	13 (100)	0.049

ECG: Electrocardiogram; QTc: Corrected QT interval; n: number of patients; SD: Standard deviation.

### 3.2 QTc monitoring in COVID-19 patients

**Figure 3:**
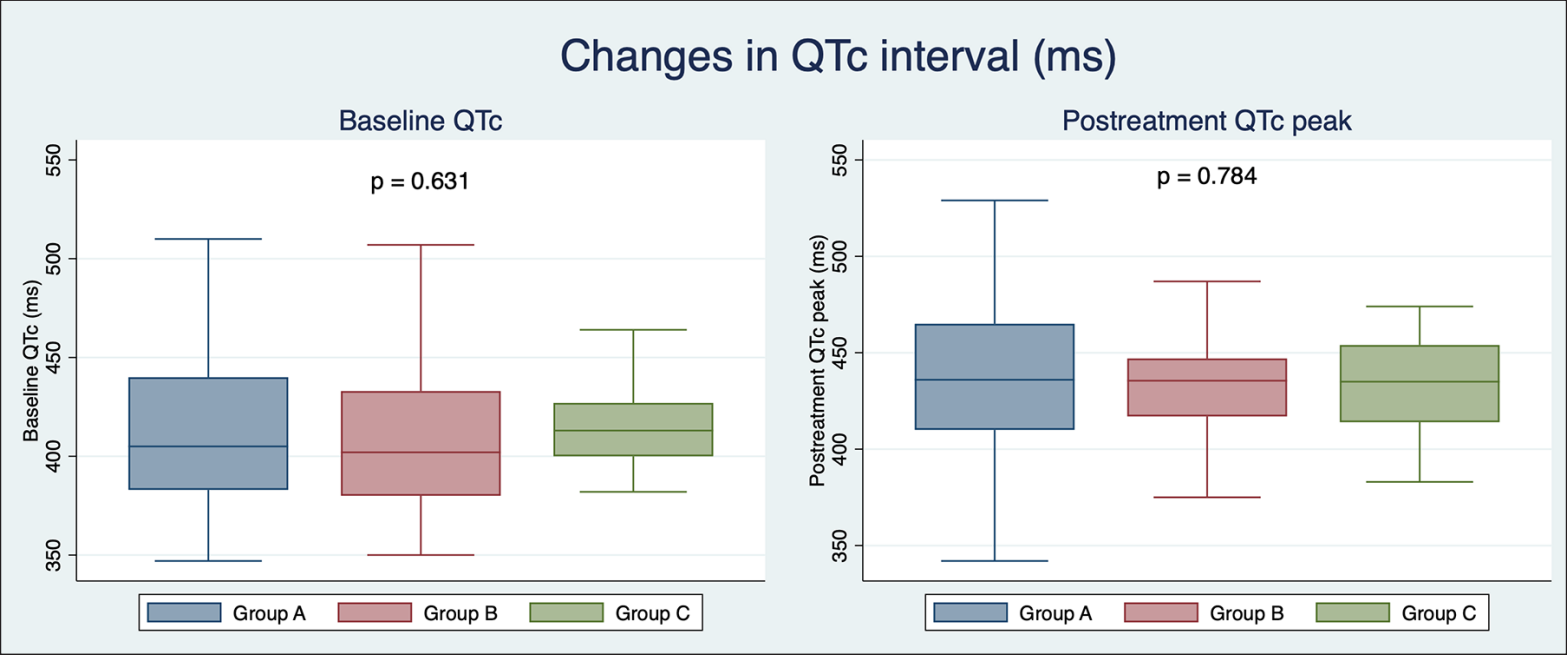
In-hospital QTc changes from baseline to peak during COVID 19 treatment documented with 12-lead ECG and/o 6-lead handheld ECG device.

**Figure 4:**
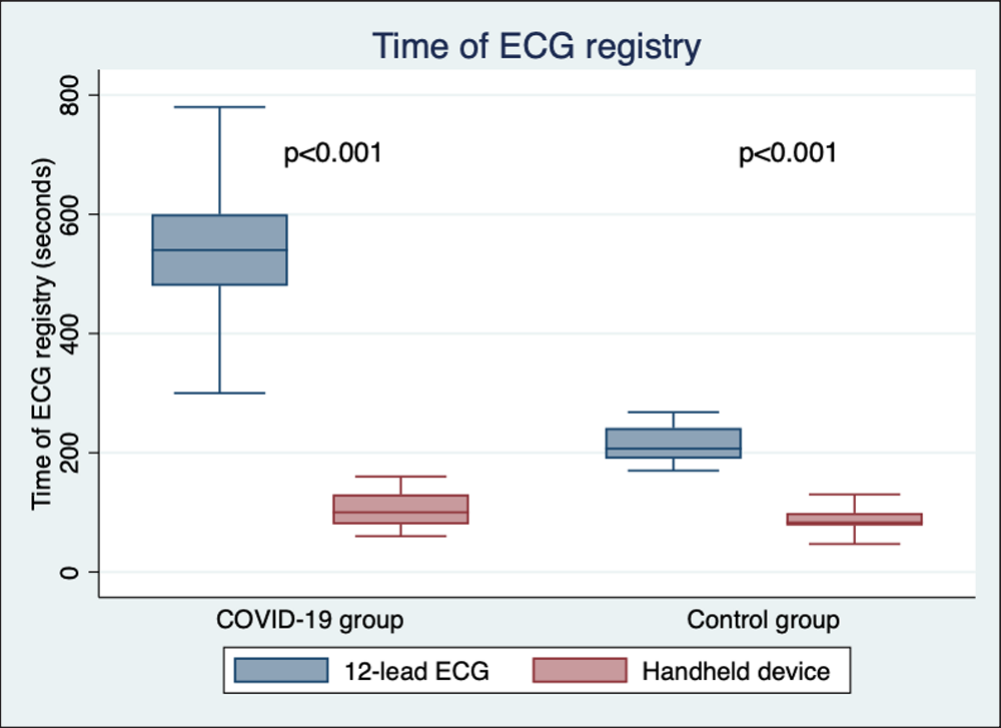
Time of ECG registry for conventional 12-lead ECG and the handheld ECG device in the control group and the COVID-19 cohort.

Table 2 summarizes ECG findings in this population. Among patients monitored with the smart device, QTc could be measured in lead II in most registries (84.5%). In the rest of the registries (15.5%), QTc was measured in another lead of the 6-lead handheld device. No significant differences were observed among the three monitoring groups regarding baseline rhythm (p = 1.0), heart rate (p = 0.749) or QTc before the administration of any COVID 19 drugs (p = 0.631). Compared to QTc at admission, QTc peak did significantly increase in all groups (12-lead ECG: 412.5 ± 34.7 vs 437.8 ± 38.6 ms, p < 0.001; 12-lead/6-lead ECG: 408.0 ± 32.9 vs 434.1 ± 28.1 ms, p < 0.001; 6-lead ECG: 416.5 ± 33.9 vs 440.2 ± 39.0 ms, p = 0.002; Figure 3). No significant differences in QTc peak (p = 0.784), QTc change from baseline (p = 0.864) or prevalence of high risk QTc prolongation (p = 0.946) were observed between the three monitoring groups. In order to know how often the smart device was not sufficient, we searched in our database for patients that were admitted in hospitalizations floors where the handheld device was fully implemented and available but were monitored with conventional 12-lead ECG. We found eight patients of the COVID 19 cohort that were not monitored with KardiaMobile despite being in a hospitalization floor with a handheld rhythm device available. This represent a 11.3% of the patients in which monitoring was attempted or performed with a handheld device (8 patients + group B + group C). Interestingly, these patients were old (85.4 ± 2.4 years) and had a high CURB65 score (3.2 ±0.5). Hypothetically, this clinical status might prevent them to collaborate to obtain an accurate registry with the smart device. In this cohort, the time needed to obtain a 12-lead ECG was significantly longer than the one needed to perform a 6-lead registry with the smart device (519.0 ± 94.1 vs 107.1 ± 37.8 seconds, p < 0.001; Figure 4).

**Table 2:** ECG findings in the three monitoring groups.

Variable	Total (n = 168)	12-lead ECG (basal and follow-up) (n = 119)	12-lead ECG (basal) and Handheld device (follow-up) (n = 50)	Handheld device (basal and follow-up) (n = 13)	p-value

Sinus rhythm, n (%)	165 (90.7)	108 (90.8)	45 (90.0)	12 (92.3)	1.000
Atrial fibrillation, n (%)	17 (9.3)	11 (9.2)	5 (10)	1 (7.7)	1.000
Pacemaker, n (%)	2 (1.1)	0 (0)	2 (4)	0 (0)	0.119
Baseline heart rate (bpm), mean ± SD	88.9 ± 19.4	89.6 ± 20.0	87.1 ± 19.3	88.5 ± 14.9	0.749
Baseline QTc (ms), mean ± SD	411.5 ± 34.0	412.5 ± 34.7	408.02 ± 32.9	416.5 ± 33.9	0.631
QTc peak (ms), mean ± SD	436.9 ± 35.9	437.8 ± 38.6	434.06 ± 28.1	440.2 ± 39.0	0.784
QTc change from baseline (ms), mean ± SD	25.4 ± 35.1	25.3 ± 37.0	26.0 ± 33.8	23.8 ± 22.7	0.864
QTc prolongation 60 (ms), n (%)	24 (13.2)	16 (13.5)	7 (14)	1 (7.7)	1.000
QTc peak 500 ms, n (%)	9 (5.0)	6 (5.0)	2 (4.0)	1 (7.7)	0.723
QTc peak 500 ms and/or QTc prolongation 60 ms, n (%)	30 (16.5)	19 (16.0)	9 (18.0)	2 (15.4)	0.946

ECG: Electrocardiogram; QTc: Corrected QT interval; bpm: beats per minute; ms: milliseconds; n: number of patients; SD: Standard deviation.

### 3.3 QTc monitoring in the control group

As a control group, both, 12- and 6-lead ECGs were obtained in 45 healthy patients (63.7 ± 18.1 years, male 56.8%). In the 6-lead registries, QTc could be measured in lead II in most cases (90.9%). Mean QTc was 411.8 ± 25.7 ms for the whole cohort with conventional 12-lead ECG and 409.1 ± 23.2 ms with the smart device, with no significant differences between both techniques (+2,7 ms 95%CI [–7,7 – 13,0], p = 0.612). Intraclass correlation coefficient for the two devices was 0.56 [0.19–0.76], displaying a moderate reliability (Supplementary Figure 1). In this setting, all the recordings but one unreadable 6-lead ECG allowed QTc measurement by interpreters, with similar diagnostic feasibility of both techniques (100% vs 97.8%, p = 0.315). Registration of a conventional 12-lead ECG in this cohort took significantly longer than to obtain a 6-lead ECG with the smart device (217.8 ± 34.3 vs 93.3 ± 29.7 seconds, p < 0.001, Figure 4). In addition, ECG registering with both devices took longer in the COVID 19 population compared to the control group (12-lead ECG: 519.0 ± 94.1 vs 217.8 ± 34.3 seconds, p < 0.001; 6-lead ECG: 107.1 ± 37.8 vs 93.3 ± 29.7 seconds, p = 0.028), although this increase of time was significantly higher for the conventional 12-lead ECG (301.2 ± 113.2 vs 13.8 ± 41.7, p < 0.001).

## Discussion

To the best of our knowledge this is the first prospective study addressing differences in QTc monitoring in COVID-19 patients receiving QTc-prolonging drugs between conventional 12-lead ECG and a 6-lead heart rhythm handheld device. The major findings of the present study were:

1) current COVID-19 therapies were significantly associated with a QTc prolongation regardless of the type of ECG monitoring; 2) the use of a handheld 6-lead ECG device significantly reduced the time of ECG registry for healthcare providers when compared to traditional 12-lead ECG; this time reduction was higher in the COVID-19 cohort compared to healthy volunteers; 3) 6-lead smart device showed diagnostic feasibility for QTc measurement similar to conventional 12-lead ECG, with a moderate reliability.

In the absence of strong evidence from randomized controlled trials, several therapies such as hydroxychloroquine, azithromycin and lopinavir-ritonavir have been empirically used to treat COVID-19 patients. Despite promising in vitro results reported at the beginning of the pandemic, most of these drugs have failed to demonstrate survival benefit in these patients. In addition, QTc prolongation is a common adverse effect of these drugs due to the inhibition of potassium channels that interfere with ventricular repolarization [7]. Moreover, drug induced QTc prolongation is associated with an increased risk of VA and SCD [8,9]. Therefore, and since most patients receive more than one drug potentially interfering with QTc, it seems reasonable to monitor the ECG while these therapies are being administered. Nevertheless, in the setting of COVID-19, conventional 12-lead ECG implies a prolonged and close exposure for healthcare workers and the need for in-depth disinfection after every registry, which limits its repeatability. In this regard, the use of mobile handheld ECG devices for QTc monitoring has emerged as an appealing alternative.

KardiaMobile (AliveCor, San Francisco, CA, USA) is a smart heart rhythm device that has been used for the detection of atrial fibrillation, the study of palpitations and pre-syncope, and the monitoring of heart rhythm abnormalities in patients with cardiac implantable devices with promising results [10,11,12]. Recently, the FDA approved this device for QTc measurement. Some reports have previously analyzed the accuracy of the ECG registry with this device to measure the QTc. Koltowsky, et al. compared ECG intervals between traditional 12-lead ECG and 1-lead KardiaMobile in a cohort of 100 patients [13]. In this series, measurements of non-corrected QT interval were shorter with the handheld device (393 vs 400 ms, p < 0.001). On the contrary, in a population of healthy volunteers receiving loading doses of dofetilide or sotalol, a device prototype that allowed the recording of leads I and II showed a good to very good reliability for measuring QTc [14]. More recently, Cheung, et al. compared QTc measurements between a 12-lead ECG and a handheld device displaying lead I and II and precordial leads V1 and V2 in 22 subjects. Although QTc measured with 12-lead ECG was overall significantly longer than the one obtained in the lead I or in the precordial leads on the handheld device, it did not differ from the one estimated in lead II of the smart device (+5 ms, 95%CI [–10–20], p = 0.244) [15]. These studies highlight the idea that QTc could be slightly underestimated, above all in 1-lead registries, with the handheld device and that measuring QTc interval on a multi-lead registry and, specially, in lead II, seems to be the most accurate method when using this device. It should be noted that none of these studies was performed with the currently available version of the device that allowed a 6-lead ECG. In our study, no significant differences in QTc were observed between the two techniques, although reliability in the control group was moderate. Interestingly, QTc could be measured in lead II in most of the patients monitored with the handheld device. Furthermore, the type of ECG monitoring was not related to clinical outcomes in this series and mortality tented to be higher among patients followed up with 12-lead ECG probably due to the worst clinical status of this subgroup.

In the setting of COVID-19, ECG monitoring can be really challenging since common serial ECGs pose exposure hazard to healthcare providers. Despite frequent QTc prolongation with COVID-19 therapies, reported rates of in-hospital VA and SCD are very low and, therefore, risk benefit balance of QTc monitoring in each patient should be individually evaluated [7]. In this regard, duration and distance of contact seem to play a key role for human to human transmission of the virus [16,17]. For this reason, continuous ECG monitoring might be the best alternative in this scenario as it avoids repeated exposure of healthcare workers and also provides VA detection. Nevertheless, its availability if often limited, even more in an oversaturated sanitary situation healthcare system like this. In a recent observational study that assessed QTc prolongation in 201 COVID-19 patients treated with hydroxychloroquine ± azithromycin, a Mobile Cardiac Outpatient Telemetry Patch (BioTelemetry, Malvern, PA, USA) was used in 58.2% of the cohort [18]. Similarly, Chang et al. used this telemetry device in 117 consecutive COVID-19 patients treated with hydroxychloroquine ± azithromycin [19]. In both reports, the authors concluded that telemetry allowed to reduce the exposure of healthcare providers by eliminating the need for serial ECGs. In our study, time of ECG recording was significantly shorter with the use of KardiaMobile-6L, reducing the need of close contact. Moreover, this discrepancy was higher in the COVID-19 cohort compared to the control group, probably due to a longer time for ECG acquisition and device disinfection. Given its lower cost and its potential use with different patients as opposed to continuous monitoring, this kind of ECG smart devices may offer an enough accurate registry for QTc measurement reducing the exposure time of healthcare providers compared to conventional ECG.

This study has several limitations inherent to its observational nature. First, sample size is small, and patients were enrolled in one single center so that our results should be interpreted with caution. Second, the absence of a direct and simultaneous comparison of QTc measurements between the smart device and conventional 12-lead ECG in the same patient in the COVID-19 cohort. Although this would have provided a stronger evidence, the increase in time of exposure for the healthcare providers to the virus was ethically unacceptable. In this regard, we performed a contemporary comparison between these two techniques in a healthy control cohort showing good feasibility of QTc estimation on handheld ECG with moderate reliability compared to conventional 12-lead ECG. Finally, given unavailability of continuous ECG monitoring in conventional hospitalization wards, accurate incidence of VA or SCD potentially associated to QTc prolongation in this scenario was not addressed.

## Conclusion

In conclusion, QTc monitoring with KardiaMobile-6L was feasible in non-severely ill conscious patients with COVID-19 receiving QTc interfering drugs. No significant differences were found in QTc measurements between the 6-lead handheld ECG and the conventional 12-lead registry, with a moderate reliability between both techniques. ECG registration time was significantly lower with the smart device, which may offer an important advantage for prevention of virus dissemination among healthcare providers in this scenario.

## Highlights

– QTc monitoring with a handheld device is feasible in non-severely ill conscious patients with COVID-19 receiving QTc interfering drugs and comparable to 12-lead ECG measurements.
– ECG registration time is lower with the handheld device, reducing exposure to healthcare providers.

## Data Accessibility Statement

The data that support the findings of this study are openly available in ‘Research Square’ at http://doi.org/10.21203/rs-38108/v1.

## Additional Files

The additional files for this article can be found as follows:

10.5334/gh.916.s1 Supplementary Figure 1. Bland-Altman Plot comparing QTc intervals measured using 12-lead ECG and 6-lead handheld ECG device. The variability between the two methods was 5.6%.
