# Supplementary figures and images for: QT Interval Monitoring with Handheld Heart Rhythm ECG Device in COVID-19 Patients
Source: Glob Heart. 2021 Jun 8;16(1):42. doi: 10.5334/gh.916 (PMC8195254; doi:10.5334/gh.916)

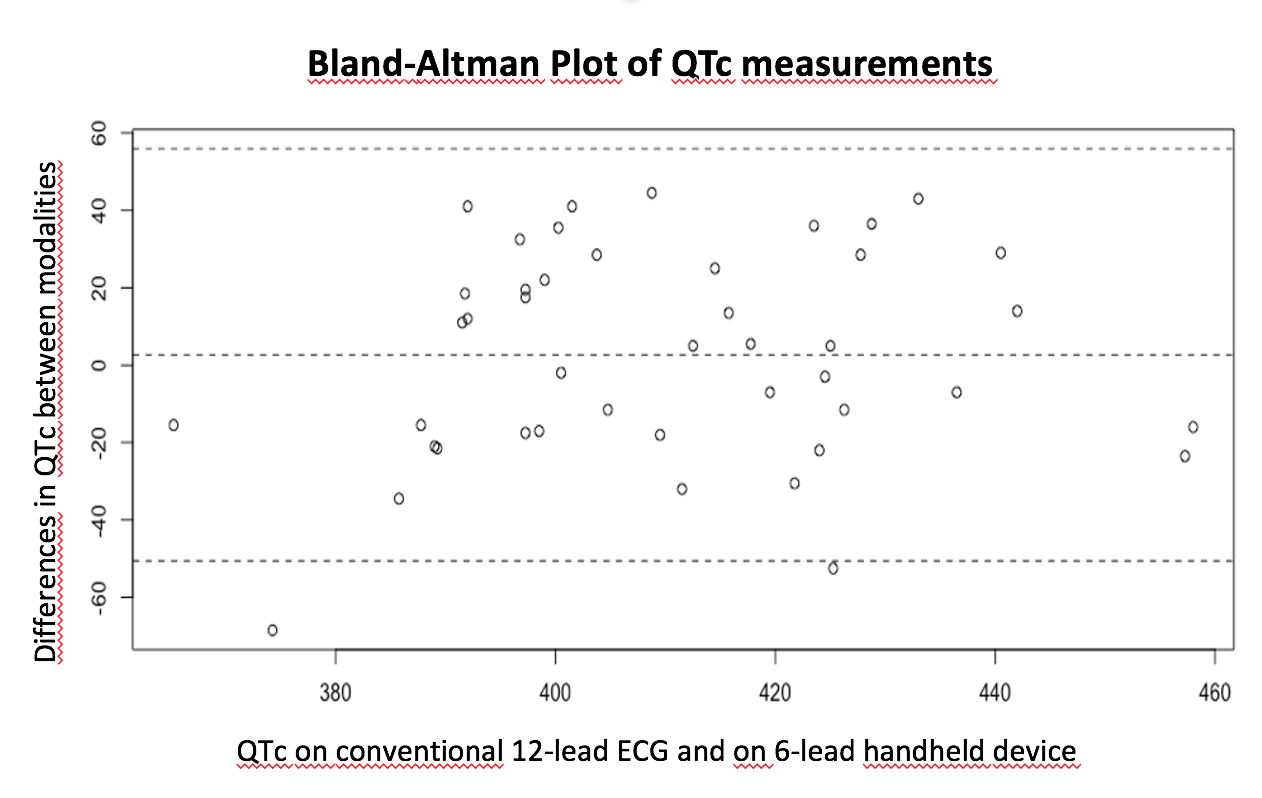

Supplement: Supplementary Figure 1. — Bland-Altman Plot comparing QTc intervals measured using 12-lead ECG and 6-lead handheld ECG device. The variability between the two methods was 5.6%. [file gh-16-1-916-s1.png]
